# Characterization of 1-3 Piezoelectric Composite with a 3-Tier Polymer Structure

**DOI:** 10.3390/ma13020397

**Published:** 2020-01-15

**Authors:** Ruiqing Sun, Likun Wang, Yanjun Zhang, Chao Zhong

**Affiliations:** 1 Beijing Key Laboratory for Sensor, Beijing Information Science & Technology University, Beijing 100192, China; sunruiqing678@163.com (R.S.); zclovelxm@163.com (C.Z.); 2 Beijing Key Laboratory for Sensor, School of Electronic Engineering, Beijing University of Posts and Telecommunications, Beijing 100876, China; fancynec@163.com

**Keywords:** 1-3 piezoelectric composite, 3-tier polymer structure, finite element method, electromechanical coupling factor, characteristic impedance

## Abstract

In order to boost the electromechanical coupling factor and decrease the characteristic impedance, a 1-3 piezoelectric composite with a 3-tier polymer structure was designed and fabricated, in which epoxy resin constitutes the middle layer and silicone rubber is used to clamp the epoxy. The effective parameters of the composite, such as resonant frequency, electromechanical coupling factor, and characteristic impedance, were studied by the finite element method and experiment. The experimental results indicate that the electromechanical coupling factor of the composite is enhanced by 8.4% and the characteristic impedance is decreased by 52.8%, compared with the traditional 1-3 ceramic/epoxy composite.

## 1. Introduction

In 1880, Pierre and Jacques Curie (the Curie brothers) discovered the piezoelectric effect (or the direct piezoelectric effect) in single crystal quartz. The piezoelectric effect is the phenomenon that certain materials produce electric charges on their surfaces as a result of applying external force, and the induced charges are proportional to the external force. Materials showing the piezoelectric effect also conversely have a geometric strain proportional to an applied electric field, which is called the converse piezoelectric effect, discovered by Gabriel Lippmann in 1881 [1]. Since the Curie brothers uncovered the piezoelectric effect, the development of piezoelectric materials can be summarized into four stages, namely single-crystal quartz, single-crystal Rochelle salt, barium titanate (BT) ceramics and lead zirconate titanate (PZT) ceramics [2].

In 1916, Dr Paul Langevin succeeded in inventing the piezoelectric transducer with single-crystal quartz [2]. His transducer received the echo from the sea floor and detected an armor plate 200 m away. It was the first time that human beings had used an echo to detect underwater targets, which was of great significance in the history of modern sonar development [3]. However, due to the low electromechanical coupling factor of single crystal quartz, Langevin’s transducer had serious defects, low mechanical underwater transmitting power, low receiving capability, and narrow bandwidth [1]. In order to overcome these drawbacks, researchers began using single-crystal Rochelle salt to make transducers. In 1919, the first Rochelle salt electroacoustic device was introduced, but single- crystal Rochelle salt tends to be degraded by humidity since it is water-soluble. After World War I, many researchers aimed to discover alternative piezoelectrics to Rochelle salt with better stability and reliability. In the following 20 years, pyroelectric and ferroelectric crystals were studied. In 1943, the discovery of barium titanate (BT) ceramics marked the development of piezoelectric ceramics from single crystals to the new fields of polycrystals [4]. Although barium titanate (BT) ceramic has a reasonably high electromechanical coupling factor and non-water solubility, it still has bottlenecks. First, under room temperature or operating temperature the barium titanate (BT) ceramics has a large temperature factor of the electromechanical parameters because of the second phase transition (from tetragonal to rhombohedral). In addition, it has an aging effect due to the low Curie temperature (phase transition from cubic to tetragonal) around only 120 °C [1]. In order to decrease the second transition temperature and to increase the Curie temperature of the barium titanate (BT) ceramics, various ion replacements, such as Pb and Ca, were studied. In 1954, Bernard Jaffe discovered the superior piezoelectricity of the lead zirconate titanate (PZT) ceramics, which triggered a new era of piezoelectric material [5]. In the 1970s, researchers began to study the relaxor ferroelectric single crystal materials. In 1997, research made a breakthrough and a new type of relaxor ferroelectric single crystal was successfully prepared, lead magnesium niobate–lead titanate (PMNT) and lead bismuth zincate–lead titanate (PZNT), which had a piezoelectric factor 3–6 times that of conventional lead zirconate titanate (PZT) ceramics [2]. At present, PZT series piezoelectric ceramics are widely used. The main characteristics are excellent mechanical and electrical properties, easy forming, controllable polarization direction, etc., and the disadvantages are high density, high characteristic impedance, and poor matching effect with water sound [2].

In 1978, Newnham et al. put forward the idea of the PZT-polymer composite, and it has achieved remarkable achievements since then. The piezoelectric composite is composed of a piezoelectric phase and a polymer phase, with a certain connection mode, a certain volume or mass ratio, and a certain spatial geometric distribution. In the piezoelectric composite, a piezoelectric ceramic with high piezoelectric properties is generally selected as the piezoelectric phase, which is usually lead zirconate titanate (PZT), lead titanate (PT) or doped lead titanate, and the polymer phase is generally epoxy resin. The components of the piezoelectric composite are self-connected in dimensions 0, 1, 2, 3. If the composite is composed of two phases, there are 10 combinations, namely 0-0, 0-1, 0-2, 0-3, 1-1,1-2,1-3, 2-2, 2-3, 3-3 [6]. The first number represents the connected dimension of the piezoelectric phase and the second represents the connected dimension of the polymer phase, which is internationally recognized [7].

There are many manufacturing techniques to produce piezoelectric composite, including the rod placement technique, the dice-fill technique, ultrasonic cutting, injection molding, lost mold, laser machining, co-extrusion, tape lamination, and fiber insertion methods [8]. Due to the simple process and design flexibility, currently the most widely accepted method for the fabrication of piezocomposite is the dice-fill technique, which was first reported by Savakus et al. [9]. The dice-fill method involves making a series of parallel cuts on a piece of bulk piezoelectric material with a mechanical dicing saw. Then, the material is diced in the perpendicular direction to produce posts with a rectangular cross section. The diced material is backfilled with a polymer, and the base ceramic support is removed [8].

1-3 piezoelectric composite can be widely used in underwater transducers [10,11,12,13], medical imaging applications [14,15], and nondestructive testing (NDT) or nondestructive evaluation (NDE) [16,17,18], mainly because it has the following characteristics [19]: (1) 1-3 piezoelectric composite has a relatively low acoustic impedance, which means it is easy to find sound absorbing material as a backing. (2) The mechanical quality factor Q of 1-3 piezoelectric composite is relatively low, which is suitable for making a broadband narrow pulse transducer. (3) The dielectric constant of 1-3 piezoelectric composite is relatively low, which gives it a relatively small static capacitance. The transducer made of 1-3 piezocomposite has high input impedance, so it has a high receiving voltage sensitivity. (4) 1-3 piezoelectric composite has a high hydrostatic piezoelectric constant, which is suitable for preparing hydrophones. (5) Due to the excellent flexibility of the polymer, 1-3 piezoelectric composite can be made into the special shapes to meet the special requirements. (6) The distribution of piezoelectric phase is controllable, so that the radiation sound field also can be controlled.

Due to the limitation of Young’s modulus of epoxy resin, the electromechanical coupling factor of conventional 1-3 ceramic/epoxy composite can only reach 0.6 approximately [20]. Many experts are working to improve the electromechanical coupling factor of piezoelectric composite. Qin et al. fabricated a 1-1-3 piezoelectric composite based on relaxor ferroelectric single crystal, which showed an electromechanical coupling factor of 0.89 [21]. However, as the price of ferroelectric single crystals are so expensive its application is limited. Therefore, several researchers prepared a 1-3 piezoelectric composite with other flexible polymers and increased its electromechanical coupling factor to about 0.68 [22]. Lee et al. find that the lower the Young’s modulus of the polymer, the higher the electromechanical coupling factor of the composite [23], whereas, too low a Young’s modulus makes the composite susceptible to deform. Besides, the characteristic impedance of the composite affects the efficiency of acoustic wave transmission. At the nonconventional approach there are also reactance-piezoelectrical effects which can change the characteristic impedance and basic properties near the serial resonance of the piezoelectric materials such as reported [24,25,26,27]. Since the characteristic impedance of the usual acoustic propagation medium, such as water, human tissue, air, etc., is relatively small (1.5 MRayls), the closer the characteristic impedance of the composite material is to them, the more efficiently the acoustic energy can be radiated [1].

In order to improve the electromechanical coupling factor of the composite as well as to give it less tendency to deform, and decrease the characteristic impedance, a composite with a 3-tier polymer structure was designed and prepared. The epoxy resin is located in the center layer of the polymer phase, providing support for the composite to resist deformation, while silicone rubber is put on the upper and lower layers, which reduces the loading effects on the piezoelectric phase [21]. In addition, the influence of the silicone rubber and piezoelectric ceramic on the characteristic impedance of the composite was also analyzed. A larger electromechanical coupling coefficient means a higher electromechanical conversion efficiency, and a lower characteristic impedance can reduce the energy loss of sound waves during radiation and reception. It is expected that from our work, a piezoelectric composite with a larger electromechanical coupling factor and a lower characteristic impedance can be found with a certain volume fraction of silicone rubber and piezoelectric ceramic.

## 2. Structure of the 1-3 Piezoelectric Composite with 3-Tier Polymer Structure

**Figure 1:**
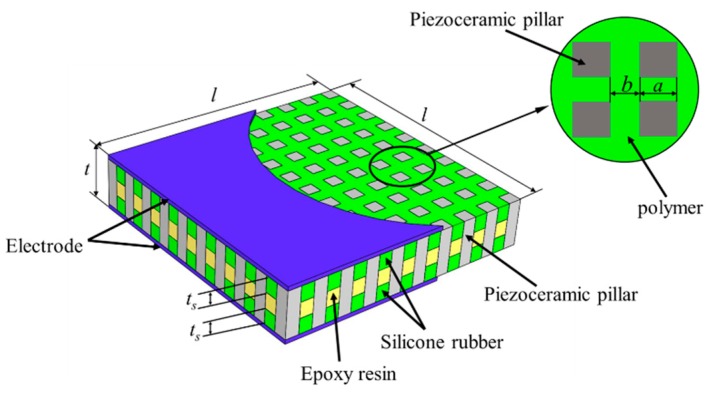
Structure of the 1-3 piezoelectric composite with 3-tier polymer structure.

As shown in Figure 1, the 1-3 composite with the 3-tier polymer structure is composed of 1-D (one-dimensional) connected piezoceramic pillars, 3-D (three-dimensional) connected polymers (epoxy resin and silicone rubber), and electrodes. The piezoceramic pillars are arranged periodically and the polymer phases have a 3-tier structure, in which the middle layer is epoxy resin while the upper and lower layers are filled with silicone rubber. In Figure 1, l and t represents the width and thickness of the composite, respectively. Moreover, a and b, refer to the width of the piezoceramic pillar and polymer. For silicone rubber, the thickness of each layer of is equal, expressed in t_s_. So the thickness of epoxy resin is t-2t_s_. The volume fraction of piezoelectric ceramic in composite v_c_ can be expressed as a^2^/(a + b)^2^, and the volume fraction of the silicone rubber in the polymer phase v_s_ is defined as 2t_s_/t.

## 3. Finite Element Analysis of the Novel Composite

**Figure 2:**
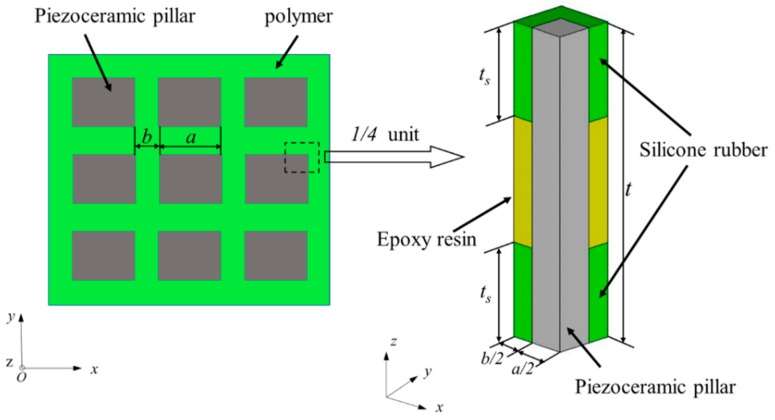
Finite element model of a 1/4 unit of the novel composite.

In order to predict the effective properties of the 1-3 piezoelectric composite with a 3-tier polymer structure, the finite element simulation method was chosen to study the resonant frequency and anti-resonant frequency, the electromechanical coupling factor, and the characteristic impedance etc. of the composite. Figure 2 depicts the finite element model of the novel composite. For the purpose of simplifying the calculation, only a 1/4 unit of the composite was established by the finite element simulation software ANSYS (15.0, ANSYS, Inc., Pittsburgh, PA, USA), and the electrode was neglected in this model for its thickness is extremely narrow. PZT-5H ceramic was selected as the piezoelectric phase while 618 epoxy resin and 704 silicone rubber were chosen as the polymer phase. Material parameters are shown in Table 1 and Table 2, respectively. The thickness of the composite t is 5 mm, while the width of the piezoelectric pillar a is 1.2 mm and the width of polymer phase b is 0.7 mm. The voltage at the top surface of the composite is 1 V and at the bottom surface is 0 V.

**Figure 3:**
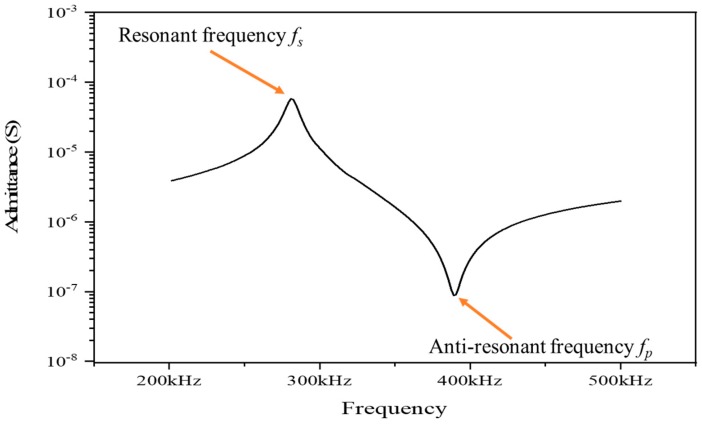
Admittance curve of the novel composite (v_s_ = 0.6, v_c_ = 0.4).

In order to study the effect of the volume fraction of silicone rubber v_s_ on the properties of the composite, harmonic response analysis was used to obtain the admittance curve of the composite, while the volume fraction of the piezoelectric ceramics v_c_ was set to 0.4. As shown in Figure 3, the resonant frequency f_s_ is the frequency at the maximum modulus point of the admittance curve and the anti-resonant frequency f_p_ appears at the minimum point.

The electromechanical coupling factor k_eff_ and sound velocity c can be calculated using (1) and (2), respectively. In addition, the equivalent density ρ and characteristic impedance z of the composite is given by (3) and (4).

k_eff_ = ((f_p_^2^ − f_s_^2^)/f_p_^2^)^1/2^ (1)

c = 2f_p_t (2)

ρ = v_c_ρ_c_ + (1 − v_c_)(v_s_ρ_s_ + (1 − v_s_)ρ_e_) (3)

where, ρ_c_ is the density of the piezoelectric phase material, ρ_e_ is the density of epoxy resin, and ρ_s_ is the density of silicone rubber.

z = ρc(4)

**Figure 4:**
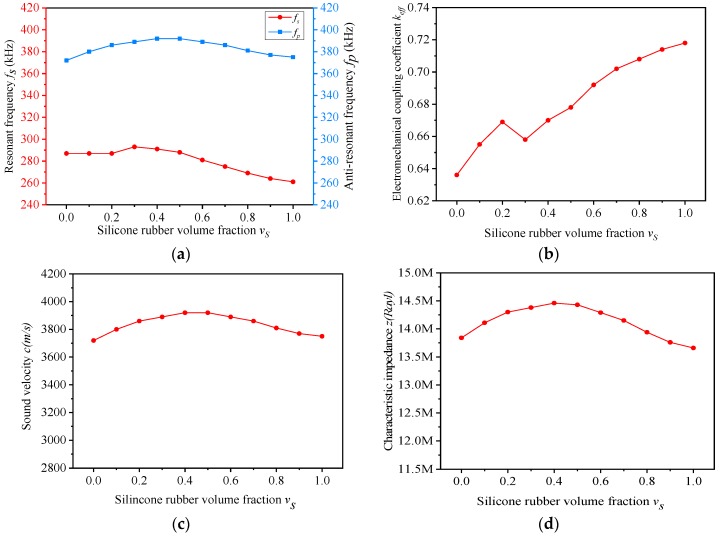
(**a**–**d**) Simulation performances of the composites with different v_s_ (v_c_ = 0.4).

The performances of the composites with different v_s_ are shown in Table 3. Accordingly, the f_s_ and f_p_ ~ v_s_, k_eff_ ~ v_s_, c ~ v_s_ and z ~ v_s_ curves are displayed in Figure 4a–d.

As shown in Figure 4a, the resonant frequency f_s_ and the anti-resonant frequency f_p_ change slightly with the increase of v_s_. The resonant frequency f_s_ is mainly determined by the thickness of the composite, which does not change in the simulation, so this is the reason for that phenomenon [28]. Furthermore, the changing trend of f_s_ and f_p_ is almost the same. When the equivalent performance parameters of the composite material are fixed, the ratio between f_s_ and f_p_ is a constant [28]. The equivalent performance parameters of the composite material do not change much in the simulation. Hence, the resonant frequency f_s_ and the anti-resonant frequency f_p_ share a similar variation tendency.

In Figure 4b, the electromechanical coupling factor k_eff_ of the composite increases with the variation of v_s_, generally. Yet, when v_s_ increases from 0.2 to 0.3, the electromechanical coupling factor k_eff_ decreases correspondingly. In this section, the increase rate of anti-resonant frequency is lower compared to that of resonant frequency, which is the reason for the decrease in k_eff_.

The curve of the sound velocity c with the increasement of v_s_ is shown in Figure 4c. As can be seen from equation (2), the sound velocity c is determined by the anti-resonant frequency and the thickness of the composite. In the simulation, the changing curve of the sound velocity c is basically consistent with the anti-resonant frequency.

Figure 4d illustrates the variation of the characteristic impedance z with the increase of v_s_. The characteristic impedance z is the product of equivalent density ρ and sound velocity c. The changing trend of sound velocity c is in accordance with the anti-resonant frequency f_p_ and the equivalent density ρ of the composite varies slightly with the increase of v_s_. Therefore, the curves of the characteristic impedance z and anti-resonant frequency f_p_ are alike.

According to the simulation data, the change of v_s_ has a great influence on the electromechanical coupling factor k_eff_ of the composite. When v_s_ is 0.6, the electromechanical coupling factor k_eff_ is 0.692, and the composite is not easily deformed under this condition. Therefore, the influence of the volume fraction of piezoelectric ceramic v_c_ on the properties of the composite was studied, when the volume fraction of silicone rubber v_s_ was 0.6.

**Figure 5:**
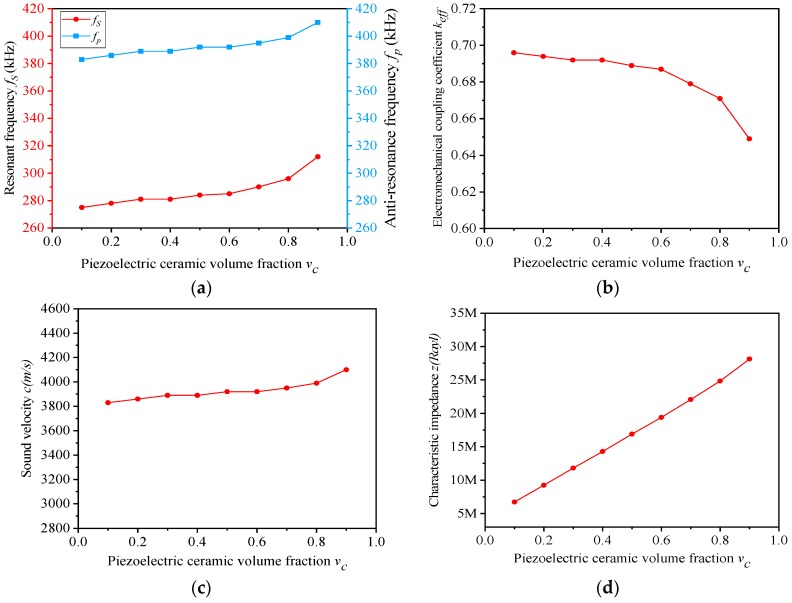
(**a**–**d**) Simulation performances of the novel composites with different v_c_ (v_s_ = 0.4).

The performances of the composites with different v_c_ are shown in Table 4. Accordingly, the f_s_ and f_p_ ~ v_c_, k_eff_ ~ v_c_, c ~ v_c_ and z ~ v_c_ curves are displayed in Figure 5a–d.

Figure 5b depicts that the electromechanical coupling factor k_eff_ showed a downward trend in general, which becomes faster and faster as v_c_ varies. When v_c_ increases, the resonant frequency f_s_ and the anti-resonant frequency f_p_ will increase simultaneously, and the growth rate of f_s_ will be slightly larger than that of f_p_. Thus, the electromechanical coupling factor k_eff_ will decrease as a whole. When v_c_ alters from 0.8 to 0.9, the rising pace of f_s_ is much larger than that of f_p_, so that k_eff_ has the largest rate of decline in this section.

Figure 5d depicts the variation of characteristic impedance z with the growth of v_c_. When v_c_ rises, the equivalent density ρ increases linearly, as can be seen from Table 4, and the fluctuation of sound velocity c is not significant. Therefore, the equivalent density ρ determines the changing trend of characteristic impedance z, which also shows a linear rise state.

## 4. Fabrication and Test

**Figure 6:**
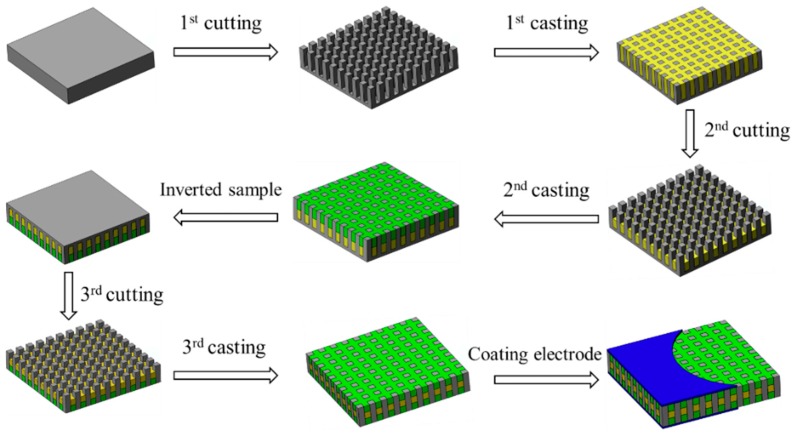
Preparation process of the novel composite.

The samples of 1-3 piezoelectric composite with 3-tier polymer structure were fabricated using the dice-and-fill method. The preparation process is shown in Figure 6. The detailed steps are as follows: First, the piezoelectric ceramic block is cut into columns arranged in arrays and a base kept. Then, the epoxy resin is filled into the cracks of the piezoelectric ceramics. Second, part of the epoxy resin is cut off along the gap between the pillars and the gap is filled with silicone rubber. Furthermore, the sample is inverted, the retained base cut into ceramic column arrays and silicone rubber filled in the gap. In the end, silver electrodes are deposited on the top and bottom by magnetron sputtering.

**Figure 7:**
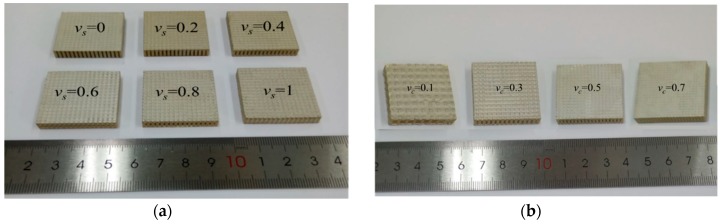
(**a**,**b**) Samples of the novel composite.

As shown in Figure 7, the composite samples are fabricated according to the preparation process. The length and width of all samples are 31 mm and the thickness is 5 mm. In Figure 7a, the volume fraction of piezoelectric ceramic v_c_ is 0.4, and the volume fraction of silicone rubber v_s_ increases from 0 to 1 with an increment of 0.2. In Figure 7b, the volume fraction of silicone rubber v_s_ is 0.6 and the volume fraction of piezoelectric ceramic v_c_ increases from 0.1 to 0.7 by 0.2. Two pieces of each type of sample are made and one of them is selected for display. The samples are measured by Impedance Analyzer (4294A, Agilent Technologies, Inc., Santa Clara, CA, USA), and the experimental data of the samples are summarized in Table 5 and Table 6.

As can be seen from Table 5 and Table 6, the electromechanical coupling factor k_eff_ of the majority of 1-3 piezoelectric composites with 3-tier polymer structure (except the composite of v_s_ = 0 and v_s_ = 1) is greater than 0.64, which indicates that the 3-tier polymer structure is beneficial in improving the electromechanical coupling factor of piezoelectric composites. In the meantime, the composite with lower characteristic impedance can be acquired, when v_c_ is less than 0.3.

**Figure 8:**
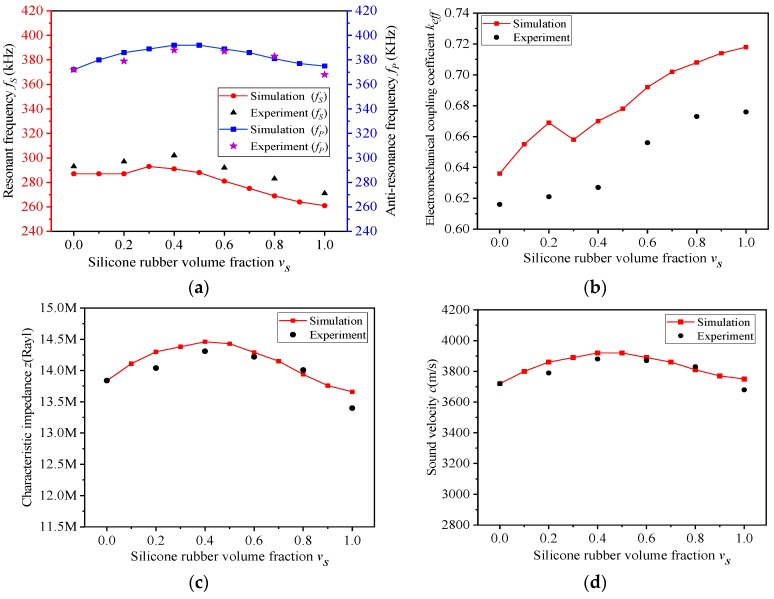
(**a**–**d**) Experiment performance of composite with different v_s_ (v_c_ = 0.4).

When v_s_ varies, the comparison between experiment and simulation are shown in Figure 8, in which the experimental data agree well with the simulation results. There are some errors between the experimental results and the simulation data, because of the difference of material parameters between experiment and simulation.

As shown in Figure 8b, when the volume fraction of silicone rubber v_s_ increases, the electromechanical coupling factor of the new composite will also increase accordingly. When v_s_ is 0.6, the electromechanical coupling factor of the advanced composite is 0.656, enhanced by 6.4%, compared with traditional 1-3 ceramic/epoxy composite (0.616). When v_s_ > 0.6, the electromechanical coupling factor k_eff_ of the composite will more than 0.67, enhancing by 8.7%. These data are calculated from Equation (5).

ξ(%) = 100 × |X_1_ − X_2_|/X_1_ (5)

In Equation (5), X_1_ represents the parameters of traditional 1-3 piezoelectric composite, and X_2_ represents. The parameters of the 1-3 piezoelectric composite with 3-tier polymer structure.

**Figure 9:**
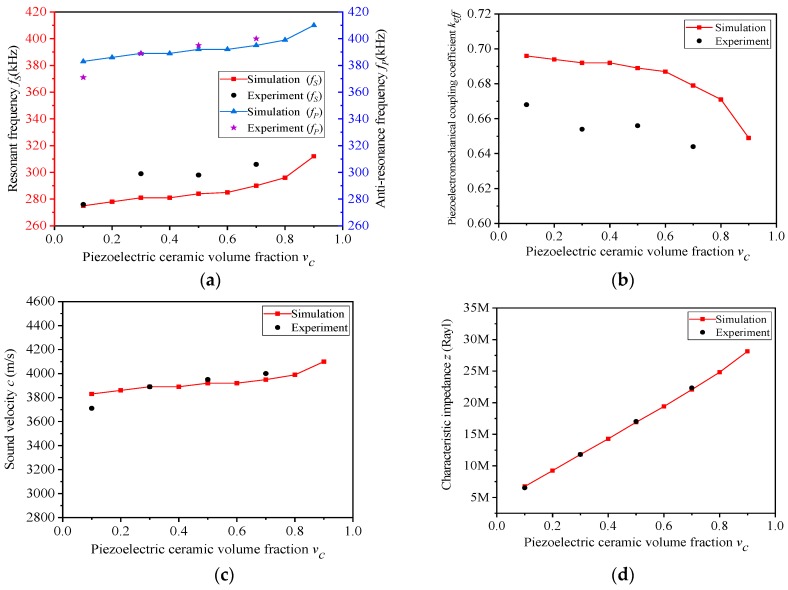
(**a**–**d**) Experimental performance of composite with different v_c_ (v_s_ = 0.6).

When v_c_ varies, the comparisons between experiment and simulation are shown in Figure 9, in which the experiment data also fit well with the simulation results. In Figure 9b, although the electromechanical coupling factor k_eff_ decreases with the increase of the volume fraction of piezoelectric ceramics v_c_, the k_eff_ of the composites remain at a high level, greater than 0.64, because the volume fraction of silicone rubber v_s_ in the composite remains unchanged. In Figure 9d, when v_c_ increases, both the test values of the characteristic impedance z and the simulation data show a linear upward trend. Moreover, the experiment–simulation error does not exceed 1%. When the volume fraction of ceramic v_c_ is less than 0.5, the characteristic impedance z of the composite can be kept at a comparatively low level. When v_c_ = 0.1 and v_s_ = 0.6, the characteristic impedance of the advanced composite is 6.53 MRayl declining by 52.8%, and the electromechanical coupling factor is 0.668 enhanced by 8.4%, compared with the traditional 1-3 ceramic/epoxy composite (13.84 MRayl, 0.616), which are also calculated by Equation (5).

## 5. Discussion

This paper introduces a 1-3 piezoelectric composite with a 3-tier polymer structure. Both the simulation and the experimental results show that the 3-tier polymer structure is advantageous for the composite to obtain a higher electromechanical coupling coefficient while keeping a comparatively low level of characteristic impedance. A larger electromechanical coupling coefficient means a higher electromechanical conversion efficiency and a lower characteristic impedance can reduce the energy loss of sound waves during radiation and reception.

## 6. Conclusions

In this paper, the finite element method is used to analyze the properties of the 1-3 piezoelectric composite with a 3-tier polymer structure. The simulation and experiment results show that the 1-3 piezoelectric composite with a 3-tier polymer structure is advantageous in obtaining a larger electromechanical coupling factor and a lower characteristic impedance. In order to verify the results of the finite element simulation, certain composite samples were prepared. Then, the samples were tested and the results agreed well with the simulation results. The experiment results indicate that when v_c_ is 0.1 and v_s_ is 0.6, the electromechanical coupling factor of the composite is enhanced by 8.4% and the characteristic impedance is decreased by 52.8%, compared with traditional 1-3 ceramic/epoxy composite.

## Figures and Tables

**Table 1:** Parameters of PZT-5H.

Density (kg/m^3^)	Piezoelectric Stress Constant (C/m^2^)			Dielectric Constant		Elastic Stiffness Coefficient (10^10^ N/m^2^)					
ρ_c_	e_31_	e_33_	e_15_	ε^S^_11_/ε_0_	ε^S^_33_/ε_0_	c_11_	c_12_	c_13_	c_33_	c_44_	c_66_
7500	−6.5	23.3	17	1700	1470	12.6	7.95	8.41	11.7	2.3	2.35

**Table 2:** Parameters of polymer.

Polymer	Density (kg/m^3^)	Young’s Modulus (N/m^2^)	Poisson’s Ratio
618 epoxy resin	1200	6.3 × 10^9^	0.3
704 silicon rubber	1070	2.55 × 10^9^	0.49

**Table 3:** Simulation data with different v_s_ (v_c_ = 0.4).

Silicone Rubber Fraction v_s_	Resonant Frequency f_s_ (kHz)	Anti-Resonant Frequency f_p_ (kHz)	Electromechanical Coupling Factor k_eff_	Sound Velocity c (m/s)	Density ρ (kg/m^3^)	Characteristic Impedance z (MRayl)
0	287	372	0.636	3720	3720	13.84
0.1	287	380	0.655	3800	3712.2	14.11
0.2	287	386	0.669	3860	3704.4	14.3
0.3	293	389	0.658	3890	3696.6	14.38
0.4	291	392	0.67	3920	3688.8	14.46
0.5	288	392	0.678	3920	3681	14.43
0.6	281	389	0.692	3890	3673.2	14.29
0.7	275	386	0.702	3860	3665.4	14.15
0.8	269	381	0.708	3810	3657.6	13.94
0.9	264	377	0.714	3770	3649.8	13.76
1	261	375	0.718	3750	3642	13.66

**Table 4:** Simulation data with different v_c_ (v_s_ = 0.6).

Piezoceramic Fraction v_c_	Resonant Frequency f_s_ (kHz)	Anti-Resonant Frequency f_p_ (kHz)	Electromechanical Coupling Factor k_eff_	Sound Velocity c (m/s)	Density ρ (kg/m^3^)	Characteristic Impedance z (MRayl)
0.1	275	383	0.696	3830	1759.8	6.74
0.2	278	386	0.694	3860	2397.6	9.25
0.3	281	389	0.692	3890	3035.4	11.81
0.4	281	389	0.692	3890	3673.2	14.29
0.5	284	392	0.689	3920	4311	16.9
0.6	285	392	0.687	3920	4948.8	19.4
0.7	290	395	0.679	3950	5586.6	22.07
0.8	296	399	0.671	3990	6224.4	24.84
0.9	312	410	0.649	4100	6862.2	28.14

**Table 5:** Experiment data with different v_s_ (v_c_ = 0.4).

Silicone Rubber Fraction v_s_	Resonant Frequency f_s_ (kHz)	Anti-Resonant Frequency f_p_ (kHz)	Electromechanical Coupling Factor k_eff_	Sound Velocity c (m/s)	Characteristic Impedance z (MRayl)
0	293	372	0.616	3720	13.84
0.2	297	379	0.621	3790	14.04
0.4	302	388	0.627	3880	14.31
0.6	292	387	0.656	3870	14.22
0.8	283	383	0.673	3830	14.01
1	271	368	0.676	3680	13.4

**Table 6:** Experiment data with different v_c_ (v_s_ = 0.6).

Piezoceramic Fraction v_c_	Resonant Frequency f_s_ (kHz)	Anti-Resonant Frequency f_p_(kHz)	Electromechanical Coupling Factor k_eff_	Sound Velocity c (m/s)	Characteristic Impedance z (MRayl)
0.1	276	371	0.668	3710	6.53
0.3	294	389	0.654	3890	11.81
0.5	298	395	0.656	3950	17.03
0.7	306	400	0.644	4000	22.35
